# Identification of AaAtg8 as a marker of autophagy and a functional autophagy-related protein in *Aedes albopictus*

**DOI:** 10.7717/peerj.5988

**Published:** 2018-11-21

**Authors:** Jialu Qiao, Dandan Zhang, Yu Wang, Xiaomei Li, Shengya Wang, Qingzhen Liu

**Affiliations:** State Key Laboratory of Virology, Modern Virology Research Center, College of Life Sciences, Wuhan University, Wuhan, Hubei, China

**Keywords:** *Aedes albopictus*, Autophagy, AaAtg8

## Abstract

*Aedes albopictus* is a primary vector of hundreds of pathogens. Strong environmental adaptability and extensive global distribution of *Aedes albopictus* make it a severe threat to human health. Autophagy is a cellular process involved in maintenance of cellular homeostasis and recirculation of cytoplasm to generate macromolecule constituents and energy under different stress conditions. Many autophagy-related (Atg) proteins have been identified in yeast and were found in various organisms subsequently, indicating that the basic mechanism of autophagy is well conserved in eukaryotes. Among all Atg proteins, Atg8 plays important roles in autophagy and is widely used as a marker to monitor autophagic activity in yeast, *Drosophila*, nematodes, zebrafish and mammals. By now, Atg proteins in *Aedes albopictus* have not been reported yet and the autophagy pathway in* Aedes albopictus* remains unclear. This study identified a homolog of Atg8 from *Aedes albopictus* and named it AaAtg8. Sequence analysis revealed that AaAtg8 was highly conserved in the Atg8 family. This work proved that AaAtg8 was a functional Atg protein of *Aedes albopictus* and expressed during developmental and adult stages of *Aedes albopictus*. Moreover, the study also established the basic methods for autophagy study in C6/36 cells. First, it was proved that both rapamycin and starvation were applicable ways to induce autophagy in C6/36 cells, and that 3-methyladenine and chloroquine could be used to inhibit early and late stages of autophagy in C6/36 cells, respectively. Second, the results in this study showed that monodansylcadaverine staining could be used to detect autophagy in C6/36 cells. Additionally, the study revealed that the level of autophagy in C6/36 cells could be monitored by the turnover assay of AaAtg8 or fluorescent AaAtg8. Taken together, this study identified AaAtg8, the first reported Atg protein in *Aedes albopictus*. It also provided useful methods for studying autophagy in *Aedes albopictus*. To our knowledge, this is the first work about autophagy in *Aedes albopictus*.

## Introduction

Autophagy, as a conserved cellular recycling pathway, is crucial for adaptation of the organisms to changing nutrient conditions and maintaining cellular homeostasis by removing redundant proteins and damaged organelles. Autophagy takes part in several physiological processes such as lifespan, development and differentiation (Levine & Klionsky, 2004; Cecconi & Levine, 2008). In addition, it can serve as a cellular defense mechanism to prevent the infection of certain viruses, parasites and pathogenic bacteria. Depending on the pathway to deliver the cargo, autophagy can be subdivided into macroautophagy, microautophagy and chaperone mediated autophagy (CMA) (Klionsky, 2005). During microautophagy process, certain substances of cytoplasm are transferred into the lysosomal lumen by direct invagination of the lysosomal membrane (Li, Li & Bao, 2012). CMA involves direct translocation of the cytosolic proteins across the lysosomal membrane, which requires substrate proteins containing a CMA-targeting motif (Cuervo, 2010). CMA has been characterized mainly in the mammalian systems, and it does not seem to function in lower organisms, such as invertebrates (Koga & Cuervo, 2011). Macroautophagy is the most prevalent form of autophagy. In the process of macroautophagy, certain proteins of cytoplasm or damaged organelles are sequestered into the double-membrane autophagosomes. Then, autophagosomes fuse with lysosomes to produce single-membrane autolysosomes, where the sequestered contents are subsequently degraded and delivered back to the cytoplasm for recycling or energy production (Feng et al., 2014).

Autophagy-related (Atg) proteins play a critical function during autophagy. More than 30 Atg proteins have been identified in yeast and higher eukaryotes. Some of the Atg proteins are responsible for the core machinery of autophagosome formation (Ohsumi, 2014). The core Atg proteins involved in macroautophagy (hereafter referred to as autophagy) can be divided into different functional groups: (1) the Atg1/ULK complex, containing Atg1, Atg11, Atg13, Atg17, Atg29 and Atg31, regulates the initiation of autophagosome formation; (2) the Atg9 system, containing Atg2, Atg9 and Atg18, functions as a carrier in supplying membrane; (3) the PI3K complex, containing Vps15, Vps30/Atg6, Vps34 and Atg14, involves in vesicle nucleation; (4) two ubiquitin-like conjugation systems: the Atg12-Atg5-Atg16 system contains Atg5, Atg7, Atg10, Atg12 and Atg16, and the Atg8 system contains Atg3, Atg4, Atg7 and Atg8, taking part in vesicle expansion (Mizushima, Yoshimori & Ohsumi, 2011; Feng et al., 2014).

Among the core Atg proteins, Atg8 play important roles in autophagy. In yeast, autophagy initiation, cargo recognition, cargo engulfment and vesicle closure are Atg8 dependent. Two major subfamilies of the sole yeast Atg8 have been found in mammals, namely, microtubule-associated protein-1 light chain 3 (LC3) and γ-aminobutyric acid (GABA)-receptor-associated proteins (GABARAP). The former consists of LC3A, B, B2 and C, whereas the latter includes GABARAP, GABARAPL1 and GABARAPL2/GATE-16 (Kabeya et al., 2004; Le Grand et al., 2011; Schaaf et al., 2016). For simplicity, “Atg8” will refer to all Atg8 family members subsequently in the text. An indispensable step for Atg8 in autophagy is its covalent conjugation to the membrane lipid phosphatidylethanolamine (PE). Atg8 is initially processed by cysteine protease Atg4 to expose the C-terminal glycine residue of Atg8 and turns into Atg8-I. Subsequently, Atg8-I is activated by Atg7 (functioning as an E1 enzyme), transferred to Atg3 (functioning as an E2 enzyme), and covalently linked to PE to form Atg8-II (Ichimura et al., 2000). The priming enzyme Atg4 also plays a role in the removal of Atg8-II from PE. Thus, Atg8 lipidation is reversible (Kirisako et al., 2000). Importantly, Atg8-II localizes to the prephagosome membranes and remains associated with mature autophagosomes even after fusion with the lysosomes (Kaufmann et al., 2014). Atg8 is the only Atg protein specifically associated with premature as well as completed autophagosome and the number of Atg8-II correlates with the amount of autophagosomes (Cheong & Klionsky, 2008; Mizushima, Yoshimori & Levine, 2010); thus, it is widely used as a specific marker to monitor autophagosomes and autophagic activity in autophagic study.

The *Aedes* mosquitoes pose a health risk to humans due to their capacity to act as disease vectors (Richards et al., 2006; Kamgang et al., 2012). They are efficient epidemic viral vectors to transmit multiple infectious viruses, including dengue virus (DENV), chikungunya virus (CHIKV) and Zika virus (Paupy et al., 2009; Waldock et al., 2013; Suwanmanee & Luplertlop, 2017). Moreover, there is no available vaccine or treatment for the diseases caused by *Aedes* mosquitoes transmitted viruses so far. Responses to the challenge of controlling these vectors are expected for us to better understand the molecular biology of these mosquitoes. Recently, it is reported that autophagy process is involved in maintaining egg maturation cycles in *Aedes aegypti* (Bryant & Raikhel, 2011). More importantly, the study published by David W. Severson’s group showed that in *Aedes aegypti* mosquito Atg proteins were upregulated in DENV-2 infection indicating a role of autophagy in virus infection (Eng, Van Zuylen & Severson, 2016). However, autophagy process in *Aedes* mosquitoes is still poorly understood. *Aedes albopictus*, as one type of *Aedes* mosquitoes, is an invasive species presenting one of the fastest spreading invasive animal species in the world. It has spread from Southeast Asia to America, Europe and Africa in the past 30 years and is becoming a major threat to the human health (Bonizzoni et al., 2013). Its rapid expansion and vectorial capacity for various arboviruses affects an increasingly larger proportion of the world population. Thus, the study of molecular mechanism in *A. albopictus* is attracting more and more attention. Recently released genome sequences of Foshan *Aedes albopictus* and Italian *Aedes albopictus* would accelerate the study of the molecular mechanisms in *Aedes albopictus* (Chen et al., 2015; Dritsou et al., 2015).

In this study, an Atg8 homolog from *A. albopictus* was identified and named *Aaatg8*. It is the first *A. albopictus* autophagy-related gene reported so far. Sequence analysis revealed that the AaAtg8 protein was highly conserved in Atg8 family. There was a generally expression of AaAtg8 during developmental and adult stages of *A. albopictus*. This work proved that AaAtg8 was a functional Atg protein of *A. albopictus* involving in autophagy process. The responses of C6/36 cells to commonly used autophagy stimuli and inhibitors were studied. Additionally, monitoring autophagy in C6/36 cells by monodansylcadaverine (MDC) staining, turnover assay of AaAtg8 and fluorescent AaAtg8 were tested. The research results demonstrated that both rapamycin (Rapa) and starvation could induce autophagy while 3-methyladenine (3-MA) or chloroquine (CQ) could inhibit early or late stage of autophagy in C6/36 cells. The data showed that MDC staining could be used to detect autophagy in C6/36 cells. This study proved that the level of autophagy in C6/36 cells could be monitored by the turnover assay of AaAtg8 or fluorescent AaAtg8. Since there are very few publications on autophagy in C6/36 cells so for, the useful information and methods provided by this study would pave the way for studying autophagy in *A. albopictus.*

## Material and Methods

### Mosquitoes and cells

*A. albopictus* was provided by Hubei Provincial Center for Disease Control and Prevention (HBCDC). *A. albopictus* mosquitoes were reared at 28 °C, 80% humidity, and a photoperiod of 16 h light: 8 h dark. Larvae and pupae were grown in cups of distilled water and fed a mixture of finely ground cat food (Nestle, Purina). Research on mosquitoes (*A. albopictus*) does not require a specific permit in Wuhan, China. All experiments were performed based on standard operating procedures in the College of Life Sciences, Wuhan University, Wuhan, China.

C6/36 cells were provided by the China Center for Type Culture Collection (CCTCC) and were maintained in a minimum essential medium (MEM; HyClone, South Logan, UT, USA) supplemented with 10% fetal bovine serum (FBS; Biological Industries, Kibbutz Beit Haemek, Israel) at 28 °C with 5% CO_2_. C6/36 cells were transfected using lipofectamine 2000 reagent (Invitrogen, Carlsbad, CA, USA) according to the manufacturer’s protocol.

### Chemical treatments and starvation treatment

For chemical treatment, C6/36 cells were treated with 3-MA (10 mM; Sigma-Aldrich, St. Louis, MO, USA), Rapa (500 nM; MedChemExpress, Shanghai, China) or CQ (300 µM; Sigma-Aldrich, St. Louis, MO, USA) for 36 h, 6 h and 36 h, respectively. When starvation treatment was performed, the C6/36 cells were maintained in Earle’s Balanced Salt Solution (EBSS; Gibco, Grand Island, NY, USA) for different time period as indicated.

### Identification and sequencing of *Aaatg8* cDNA

Using primers designed according to some Atg8 homolog genes of insects, fragments of *Aaatg8* were got from cDNA of C6/36 cells through PCR. Then, the products of PCR were cloned into pCR-II vectors (TA Cloning® Kit; Invitrogen, Carlsbad, CA, USA) and were sequenced by Sangon Biotech, Shanghai, China. The primers for rapid amplification of cDNA ends (RACE) were designed according to obtained sequences. Then, 5′ and 3′ RACE reactions were operated with SMARTer™ RACE cDNA Amplification Kit (Clontech, Mountain View, CA, USA). After purification and sequenced, 5′ and 3′ RACE reaction products were cloned into pCR-II. The open reading frame (ORF) sequence was deduced from the sequencing data. Then, the ORF fragment was amplified and cloned into pCR-II. The sequencing results from positive colonies confirmed the sequences information of previous RACE products. The involved primers are listed in Table 1.

**Table 1:** Primers used in the study.

**Primer name**	**Description**	**Primer sequence (5′–3′)**
AaAtg8-5′RACE	5′RACE primer	GGTACAGCGAGCCCATTGTTGCCG
AaAtg8-3′RACE	3′RACE primer	CGTGGGAGATAAAATCCGACGCAAATAC
AaAtg8-F	PCR primer, forward	ATGGTGAGCAAGGGCG
AaAtg8-R	PCR primer, reverse	TTACTTGTTTCCATACACATTCTC
AaS7-341-F	Real-time PCR primer, forward	GTCCACGATCCCGCACTCT
AaS7-488-R	Real-time PCR primer, reverse	GTGGTCTGCTGGTTCTTGTCC
AaAtg8-F	Real-time PCR primer, forward	CCCGTGATTGTTGAGAAAGC
AaAtg8-R	Real-time PCR primer, reverse	ATTGTTGCCGATGTTGGTGG
GFP-AaAtg8-F	Plasmid construction primer, forward	CCCTCGAGATGAAATTTCAATACAAGGAAGAAC
GFP-AaAtg8-R	Plasmid construction primer, reverse	GAAGATCTTTACTTGTTTCCATACACATTCTCAT
RFP-GFP-AaAtg8-F	Plasmid construction primer, forward	GGGGTACCATGGTGAGCAAGGGCGAG
RFP-GFP-AaAtg8-R	Plasmid construction primer, reverse	GCGTCGACTTACTTGTTTCCATACACATTC

### Construction of plasmids

The pIE-GFP-AaAtg8 plasmid was constructed by cloning the coding region of AaAtg8 into the *Xho I* and *Bgl II* sites of pIE-GFP vector. The pIE-RFP-GFP-AaAtg8 plasmid was constructed by cloning the coding region of GFP-AaAtg8 into the *Kpn I* and *Sal I* sites of pIE-RFP plasmid. The involved primers are listed in Table 1.

### Quantitative real-time PCR

Total RNA of C6/36 cells or homogenizing *A. albopictus* mosquitoes was extracted by using TRIzol regent (Invitrogen, Carlsbad, CA, USA) and used as the template for cDNA synthesis by using M-MLV Reverse Transcriptase (Invitrogen, Carlsbad, CA, USA) according to the protocol. The obtained cDNAs were used as templates for quantitative real-time PCR (qPCR) amplification using FastStart universal SYBR master (Roche, Penzberg, Germany) according to the manufacturer’s protocol. Specific gene transcription level was normalized to that of ribosomal gene *s7* (*Aas7*; GenBank: JN132168.1). Quantity values were obtained by means of the 2^−ΔΔ*Ct*^ method (Livak & Schmittgen, 2001). Three biological replicates were performed in each experiment. The involved gene-specific primers are listed in Table 1.

### Western blot analysis

Prepared cell lysates were boiled for 5 min in SDS loading buffer. Proteins were separated by SDS-PAGE and transferred to Immobilon-P membrane (Merck Millipore, Darmstadt, Germany). Membranes were blocked in Tris-buffered saline (TBS) with 5% nonfat dry milk (Sangon Biotech, Shanghai, China) and 0.1% Tween 20 (Sangon Biotech, Shanghai, China) for 1 h and then incubated with mouse anti-actin antibody (1:10,000; Proteintech, Rosemont, IL, USA), rabbit anti-AaAtg8 antibody which was antibody against GABARAP (1:1,000; MBL, Tokyo, Japan) or mouse anti-GFP antibody (1:3,000; Santa Cruz Biotechnology, Dallas, TX, USA) for 1 h. After washed three times with TBST, the membrane was incubated by an anti-rabbit or anti-mouse antiserum conjugated to horseradish peroxidase (anti-rabbit 1:5,000 and anti-mouse 1:5,000; Thermo Fisher Scientific, Waltham, MA, USA) for 1 h. Then the membrane was washed three times with TBST. After incubating the membrane with HRP substrate (Merck Millipore, Darmstadt, Germany), the bands were visualized by LAS 4000 (Fujifilm, Tokyo, Japan). The images’ brightness and contrast were adjusted using ImageJ 1.51k (National Institutes of Health, Bethesda, MD, USA), and the backgrounds were subtracted (Schindelin et al., 2012). To measure the integrated densities, the intensities of regions of interest (ROIs) were selected for all bands and normalized to the corresponding Actin.

### Fluorescence microscopy and software-aided counting

In MDC (Sigma-Aldrich, St. Louis, MO, USA) staining assay, C6/36 cells were incubated with 50 µM MDC at 28 °C for 10 min. After incubation, cells were washed three times with 0.1 M PBS (pH 7.4) and fixed with 4% paraformaldehyde in 0.1 M PBS (pH 7.4) for 10 min at room temperature. Then, cells were washed three times with PBS. Cell images were taken with an automated microscope (FV1000, Olympus, Tokyo, Japan).

In fluorescent AaAtg8 assay, after GFP-AaAtg8 or RFP-GFP-AaAtg8 was overexpressed in C6/36 cells for the time period as indicated, cells were fixed with 4% paraformaldehyde in 0.1 M PBS (pH 7.4) for 15 min at room temperature. Then, cells were washed three times with PBS and cell nuclei were counterstained with Hoechst 33342 (Sigma-Aldrich, St. Louis, MO, USA). Cell images were taken with a scanning confocal microscope (SP8, Leica, Wetzlar, Germany).

We used ImageJ 1.51k as a tool for fluorescent spots counting in cells (Schindelin et al., 2012; Guillery & August, 2002). Fluorescent spots were counted in ROIs of each C6/36 cell.

### Data analysis

Multiple-sequence alignment was performed with DNAMAN Version7 (Lynnon Corporation, San Ramon, CA, USA). Phylogenetic analysis was performed through MEGA 6 (Tamura et al., 2013). Secondary structures were predicted with ESPript3.0 (Robert & Gouet, 2014). The pictures were edited with Adobe Photoshop CC 2017 (Adobe, San Jose, California, USA). GraphPad Prism (GraphPad Software, Inc., San Diego, CA, USA) was used for plotting graphs. All data was presented as Mean ± SEM of triplicate experiments. Group comparisons were performed by *t* test or one-way ANOVA with the Duncan test with SPSS 19 system software (SPSS Inc. Chicago, IL, USA). A value of *P* less than 0.05 was considered to be significant differences.

## Results

### Sequence analysis of AaAtg8

**Figure 1:**
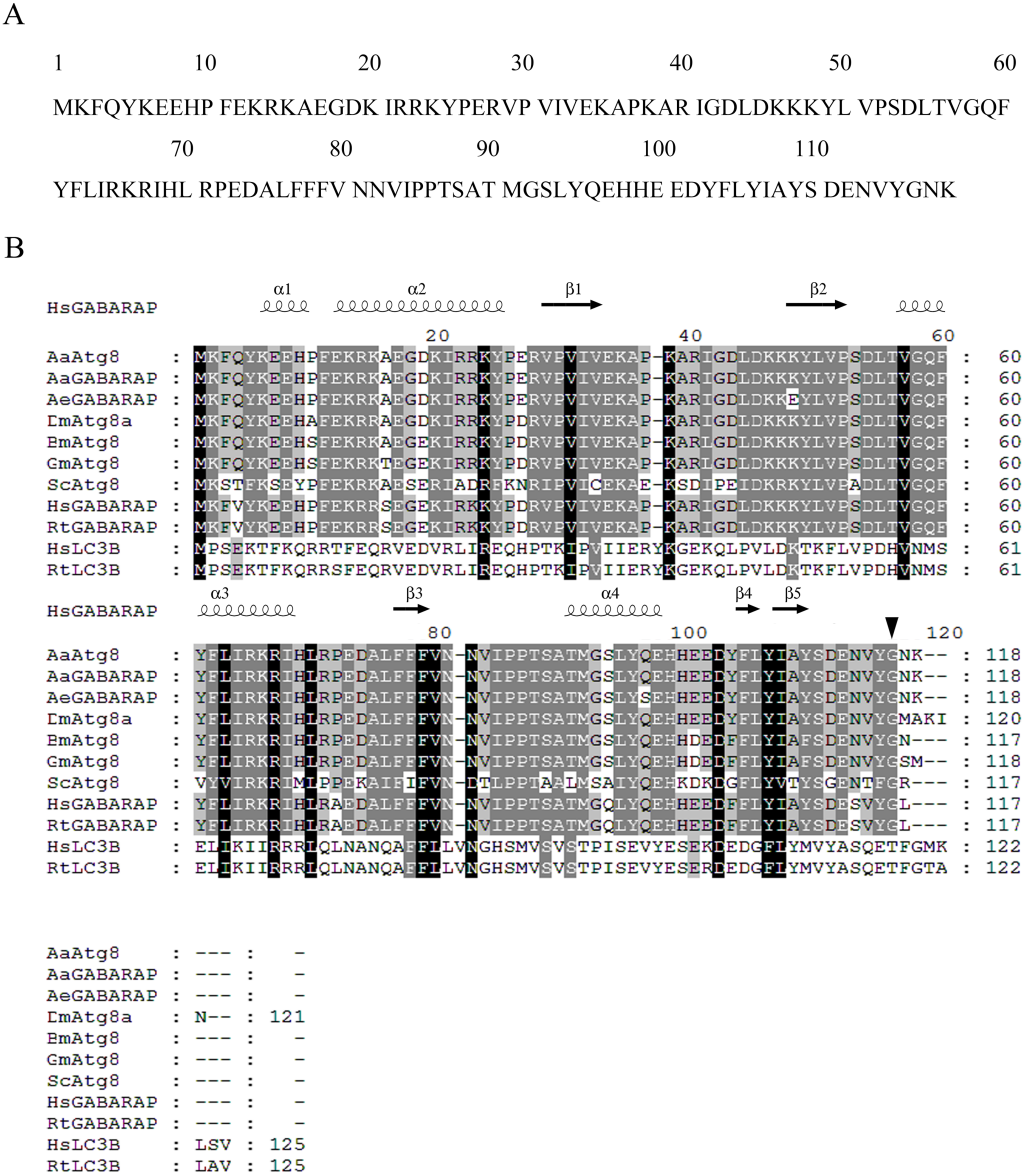
Sequence analysis of AaAtg8. (A) Predicted amino acid sequence of AaAtg8. (B) Multiple alignment of conserved domain of AaAtg8 from *A. albopictus* with other A tg8 family members from *A. aegypti* (AeGABARAP/AeAtg8), *Drosophila melanogaster* (DmAtg8), *Bombyx mori* (BmAtg8a), *Galleria mellonella* (GmAtg8), *Saccharomyces cerevisiae* (ScAtg8), *Homo sapiens* (HsGABARAP and HsLC3B) and *Rattus norvegicus* (RtGABARAP and RtLC3B). The amino acid residues identical among 10 homologs were indicated by white letters within black boxes, the amino acid residues identical between 8 homologs were indicated by white letters within dark gray boxes, and the amino acid residues identical between 6 and seven homologs were indicated by black letters within light gray boxes. Black arrow: predicted cleavage sites of Gly116 cleaved by AaAtg4. Secondary structures were predicted using ESPript3.0 based on HsGABARAP, α, α-helix; β, β-sheet.

Primers were designed according to *Aedes aegypti Atg8/GABARAP* gene (*Aeatg8/AeGABARAP*, NCBI Reference Sequence: XP_001652571.1) and cDNA of C6/36 cells as templates, partial sequence of *Aaatg8* was amplified by PCR. Based on that partial sequence information, a complete ORF fragment containing of 357 bp as well as a 5′ *UTR* of 151 bp and a 3′ *UTR* of 747 bp was obtained by 5′ and 3′ RACE reactions. The obtained ORF sequence encoded a protein containing 118 amino acids (Fig. 1A).

Multiple-sequence alignment revealed that AaAtg8 had high sequence identities with other Atg8s. The protein sequence of AaAtg8 shared 96%, 93%, 93%, 92%, 90%, 90%, 57%, 29% and 28% sequence identities with Atg8 from *A. aegypti* (AeGABARAP/AeAtg8, NCBI Reference Sequence: XP_001652571.1), *D. melanogaster* (DmAtg8a, NCBI Reference Sequence: NP_727447.1), *Bombyx mori* (BmAtg8, NCBI Reference Sequence: NP_001040244.1), *Galleria mellonella* (GmAtg8, GenBank: AFP66874.1), *Homo sapiens* (HsGABARAP, NCBI Reference Sequence: NP_009209.1), *Rattus norvegicus* (RtGABARAP, NCBI Reference Sequence: NP_742033.1), *Saccharomyces cerevisiae* (ScAtg8, NCBI Reference Sequence: NP_009475), *Homo sapiens* (HsLC3B, NCBI Reference Sequence: NP_073729.1) and *Rattus norvegicus* (RtLC3B, NCBI Reference Sequence: NP_074058.2), respectively. Then, the secondary structure was predicted with ESPript3.0 based on HsGABARAP (PDB: 1KOT) (Gouet, Robert & Courcelle, 2003). The predicted secondary structure of AaAtg8 consisted of a series of alpha helices and beta sheets that are highly conserved in other Atg8 proteins (Fig. 1B).

### Phylogenetic analysis of AaAtg8

**Figure 2:**
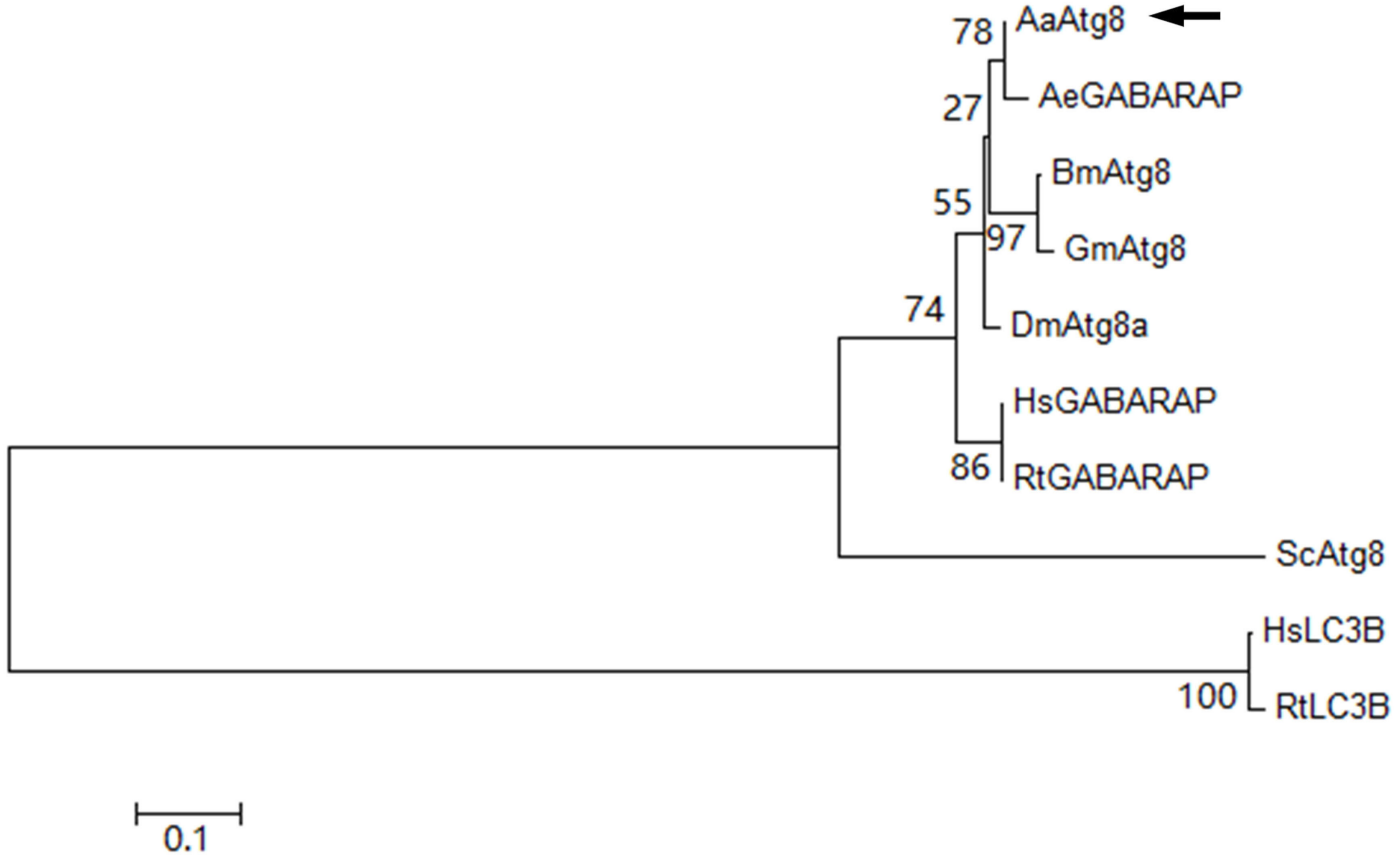
Phylogenetic analysis of AaAtg8. The predicted amino acid sequence of AaAtg8 together with nine selected Atg8 family members was aligned and the phylogenetic tree was constructed by MEGA 6 using the neighbor-joining method. The sequences include AaAtg8 from *Aedes aegypti*, AeGABARAP/AeAtg8 from *A. albopictus*, BmAtg8 from *Bombyx mori*, DmAtg8a from *Drosophila melanogaster*, GmAtg8 from *Galleria mellonella*, HsGABARAP and HsLC3B from *Homo sapiens*, RtGABARAP and RtLC3B from *Rattus norvegicus*, ScAtg8 from *Saccharomyces cerevisiae*. AaAtg8 was indicted by a black arrow.

Phylogenetic tree of AaAtg8 and nine selected Atg8 from other species was constructed on the basis of distances of amino acid sequences. The obtained dendrogram placed AaAtg8 in the insect group but separated from mammalian LC3B proteins (Fig. 2). The phylogenetic analysis also illustrated that AaAtg8 and AeGABARAP/AeAtg8 formed a single clade inside the insect group, indicating that AaAtg8 was closer to GABARAP than to LC3B.

### AaAtg8 expression profile in developmental and adult stages of *Aedes albopictus*

**Figure 3:**
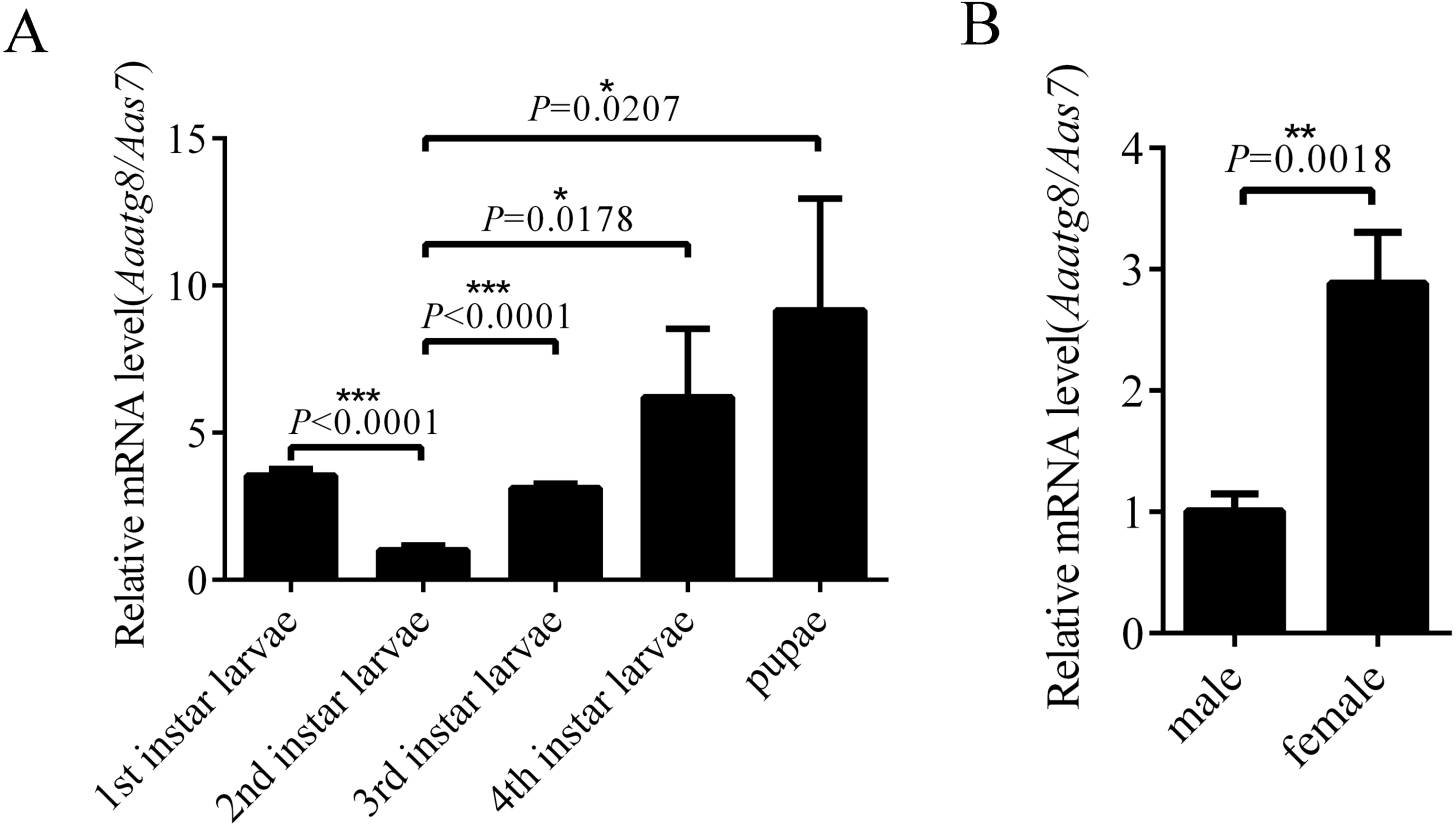
AaAtg8 expression profile in developmental and adult stages of *Aedes albopictus*. Total RNAs were prepared from 1st to 4th instar larvae, pupae, male and female adults and then subjected to qPCR analysis. The vertical axis represents the relative expression of *AaAtg8* in different developmental stages or different genders relative to housekeeping gene *Aas7*. The statistical significances were calculated by *t* test, *P < 0.05, **P < 0.01, ***P < 0.001.

In order to research the expression profile of AaAtg8, the transcription levels of *Aaatg8* in different developmental stages of *A. albopictus* were analysed by qPCR. *A. albopictus* ribosomal gene *s7* was used as a control gene. There was a ubiquitous expression of AaAtg8 in all the developmental stages. The transcription level of AaAtg8 increased progressively from 2nd instar larvae to pupae, which implied that AaAtg8 might play a role in development (Fig. 3A). Also, transcription level of AaAtg8 in female adults was about 3 times as that in male adults (Fig. 3B).

### Responses of *A. albopictus* C6/36 cells to chemicals commonly used in autophagy study

**Figure 4:**
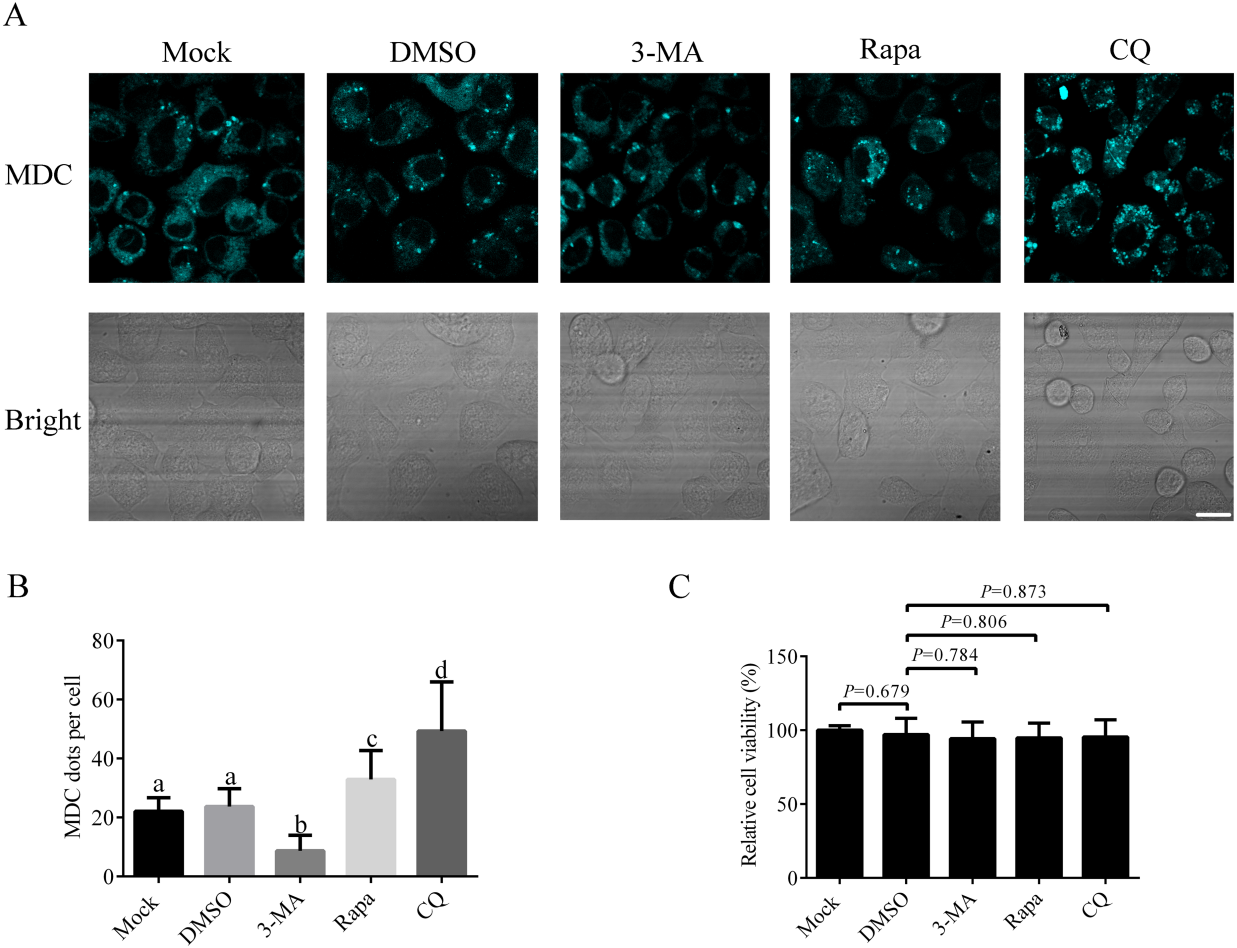
Responses of *Aedes albopictus* C6/36 cells to 3-MA, Rapa or CQ treatment. C6/36 cells were treated with 3-MA, Rapa or CQ for 36 h, 6 h and 36 h, respectively and then subjected to MDC staining. Mock and DMSO treated C6/36 cells were used as controls. After MDC staining, cells were observed under fluorescence microscope (A) and the average number of MDC-labeled vacuoles counted by ImageJ in 10 cells was graphed (B). (C) The cell viability of C6/36 cells was determined by the MTT assay after C6/36 cells were treated with 3-MA, Rapa or CQ for 36 h, 6 h and 36 h, respectively. The statistical significances were calculated by *t* test or one-way ANOVA. Different lowercase letters (a, b, c and d) indicate significant differences among experimental groups (*P* < 0.05). Scale bar: 10 µm.

Since there is no research on autophagy process in *A. albopictus* C6/36 cells published so far, it is important to establish the basic method to study autophagy in C6/36 cells. We first studied how to induce or inhibit autophagy in C6/36 cells. For that purpose, C6/36 cells were treated with 3-MA (an inhibitor that effectively blocks early stage of autophagy), Rapa (a kind of widely used autophagy inducer) or CQ (an acidification inhibitor blocking late stage of autophagy) and the effect of each treatment was evaluated by fluorescence microscopy after monodansylcadaverine (MDC) staining. Mock and DMSO treated C6/36 cells were used as controls. MDC is an acidotropic dye routinely used as an indicator in autophagy assays due to its capacity to stain acidic autolysosomes. The data showed that a few MDC fluorescent puncta were observed in mock treated C6/36 cells, indicating a basal level of autophagy in C6/36 cells. DMSO treatment did not change the number of fluorescent puncta. Compared to that in DMSO treated C6/36 cells, the number of fluorescent puncta decreased in 3-MA treated C6/36 cells, whereas increased in Rapa treated C6/36 cells. The number of fluorescent puncta in CQ treated C6/36 cells was higher than that in the DMSO or Rapa treated C6/36 cells (Figs. 4A and 4B). The above data suggested that Rapa could be used to induce autophagy, while 3-MA and CQ were effective agents to inhibit autophagy at early and late stages of autophagy in C6/36 cells, respectively (see ‘Discussion’). Moreover, to determine whether these chemicals affect the C6/36 cells viability, we performed the MTT assay. The result revealed that there were not significant effects on the viability of C6/36 cells treated with these chemicals (*P* > 0.05) (Fig. 4C).

### AaAtg8 was involved in autophagy and a useful marker for autophagy assay in C6/36 cells

**Figure 5:**
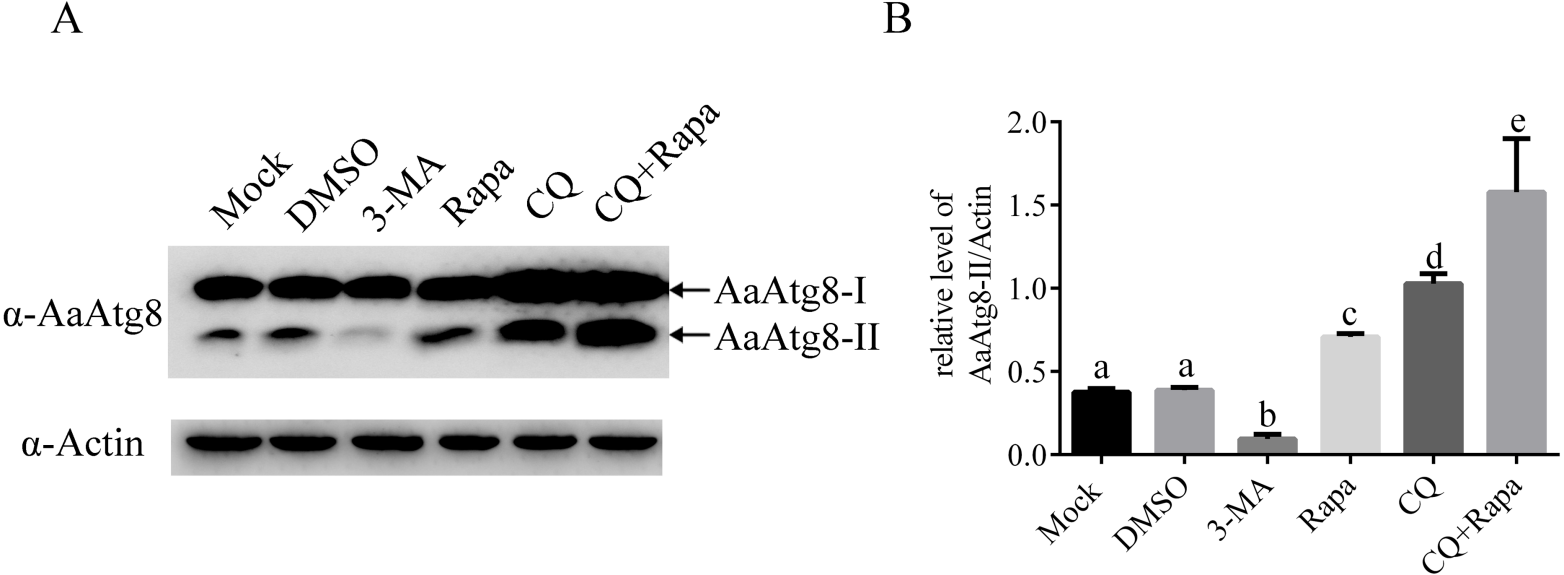
AaAtg8 was involved in autophagy and a useful marker for autophagy assay in C6/36 cells. C6/36 cells were treated with 3-MA, Rapa or CQ for 36 h, 6 h and 36 h, respectively. For Rapa plus CQ treatment, C6/36 cells were treated with CQ for 30 h then treated with Rapa for 6 h. Mock and DMSO treated C6/36 cells were used as controls. After the treatment, the levels of AaAtg8-I and AaAtg8-II were analysed by immunoblotting using antibody against AaAtg8 (A) and the relative levels of AaAtg8-II/Actin were quantified and graphed according to materials and methods (B). The normalized ratio for AaAtg8-II was calculated by dividing the mean signal intensity from three biological replicates by the mean signal intensity with Actin. The statistical significances were calculated by one-way ANOVA. Different lowercase letters (a, b, c, d and e) indicate significant differences among experimental groups (*P* < 0.05).

As described before, during the process of autophagy, Atg8-I is transformed to Atg8-II that specifically associates with premature as well as completed autophagosomes. So, Atg8-II levels correlate with the number of autophagosomes and Atg8-II is widely used as a marker to monitor autophagosomes and autophagic activity (Mizushima, Yoshimori & Levine, 2010). In order to study whether AaAtg8 could indicate autophagic level in C6/36 cells similar to other research systems, the levels of AaAtg8-I and AaAtg8-II in 3-MA, Rapa or CQ treated C6/36 cells were assessed by immunoblotting using an antibody against AaAtg8. Mock treated and DMSO treated cells were used as controls. The data showed that in mock treated C6/36 cells, high levels of AaAtg8-I and low levels of AaAtg8-II were detected, suggesting that there was a basal level of autophagy in C6/36 cells. The pattern remained unchanged in DMSO treated C6/36 cells. Compared to that in DMSO treated C6/36 cells, the levels of AaAtg8-II were decreased in 3-MA treated C6/36 cells whereas increased in Rapa treated C6/36 cells, indicating that 3-MA inhibited the early stage of autophagy and Rapa induced autophagy in C6/36 cells (see ‘Discussion’). In CQ treated C6/36 cells, levels of AaAtg8-I and AaAtg8-II were up-regulated. In C6/36 cells treated by both Rapa plus CQ, the levels of AaAtg8-II were even higher than those in C6/36 cells treated by Rapa or CQ alone (Fig. 5). These results further confirmed that Rapa could successfully induce autophagy and CQ could inhibit the late stage of autophagy in C6/36 cells (see ‘Discussion’). Taken together the above results and previous data, these findings indicated that the turnover of AaAtg8 was a useful marker of autophagy in C6/36 cells and could be used to monitor the levels of autophagy in *A. albopictus*.

Moreover, as shown in previous MDC staining assay, the number of fluorescent puncta was decreased in 3-MA treated C6/36 cells. Here, in immunoblotting analysis, the levels of AaAtg8-II were decreased in 3-MA treated C6/36 cells. Similarly, in Rapa or CQ treated C6/36 cells, the number of fluorescent puncta in MDC staining assay and the levels of AaAtg8-II in immunoblotting analysis were increased. These results proved that AaAtg8 was a functional Atg protein involving in autophagy in *A. albopictus* C6/36 cells.

### Fluorescent AaAtg8 could be used to monitor autophagy in C6/36 cells

**Figure 6:**
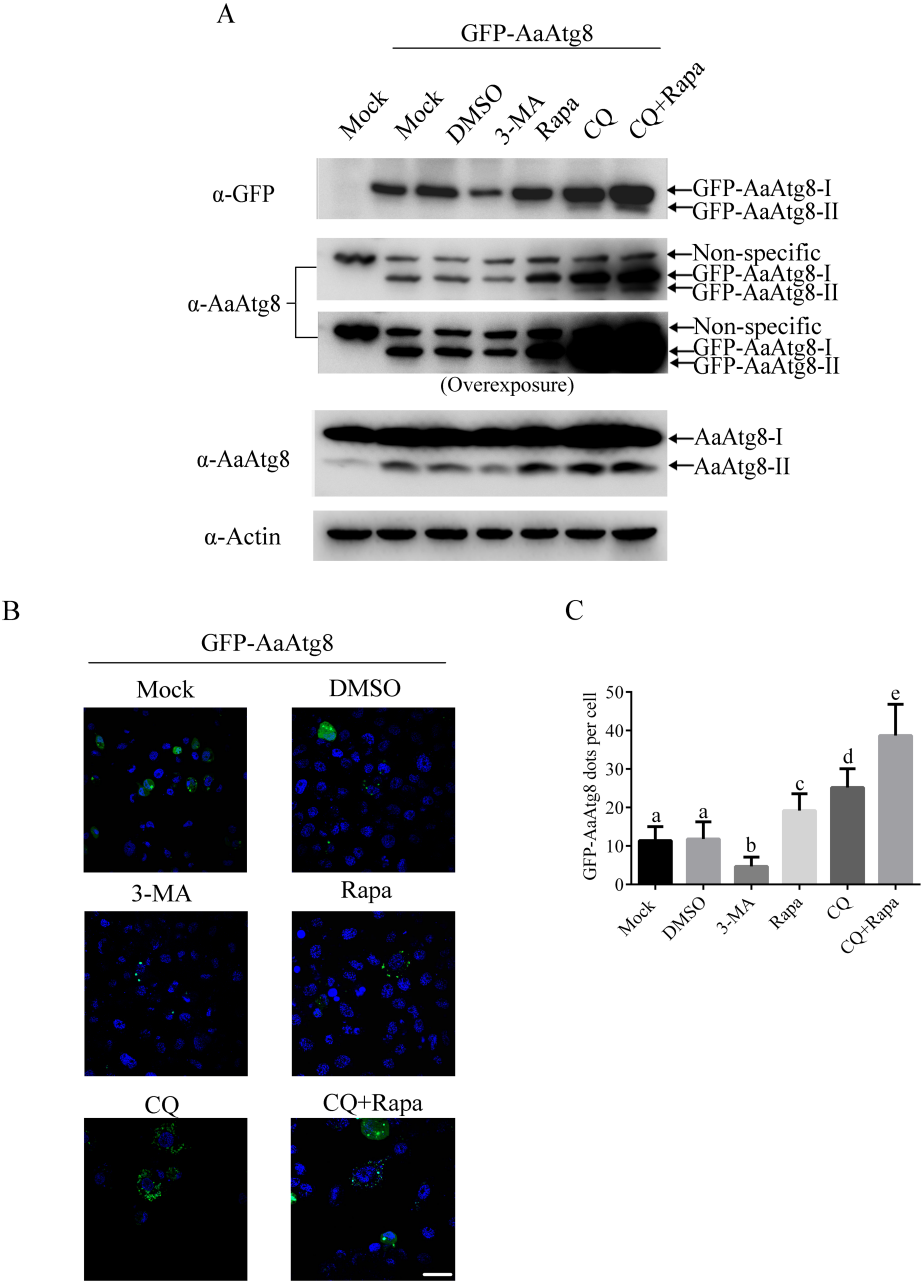
GFP-AaAtg8 could be used to monitor autophagy in C6/36 cells. C6/36 cells were transfected with GFP-AaAtg8 plasmid for 12 h and then C6/36 cells were treated with 3-MA, Rapa or CQ for 36 h, 6 h and 36 h, respectively. For Rapa plus CQ treatment, C6/36 cells were treated with CQ for 30 h then treated with Rapa for 6 h. Mock and DMSO treated C6/36 cells were used as controls. Level of endogenous AaAtg8 and GFP-AaAtg8 were analysed by immunoblotting using antibody against AaAtg8 and GFP (A). Cells were observed and cell images were taken under fluorescence microscope (B). The average number of GFP-AaAtg8 vacuoles counted by ImageJ in 10 cells was graphed (C). In (A), the second picture from the top was overexposed to form the third one from the top to show GFP-AaAtg8-II bands clearly. Hoechst 33342 was used to stain nuclear DNA. The statistical significances were calculated by one-way ANOVA. Different lowercase letters (a, b, c, d and e) indicate significant differences among experimental groups (*P* < 0.05). Scale bar: 10 µm.

When being observed under fluorescence microscopy, fluorescent Atg8 can be visualized as a diffused cytoplasmic pool when cells are under normal conditions or as punctate structures when cells undergo autophagy process. Thus, fluorescent Atg8 provides a convenient way to monitor autophagy and it has been successfully applied to study autophagy in many model organisms including *Drosophila*, nematodes, plants, zebrafish and mouse (Melendez et al., 2003; Mizushima et al., 2004; Rusten et al., 2004; Scott, Schuldiner & Neufeld, 2004; Yoshimoto et al., 2004; He et al., 2009). In order to determine whether this method could be used in C6/36 cells, GFP-AaAtg8 overexpressing C6/36 cells were treated with 3-MA, Rapa or CQ, and levels of GFP-AaAtg8 were monitored via immunoblotting and the number of GFP-AaAtg8 dots was observed by fluorescence microscopy. Mock or DMSO treated C6/36 cells were used as controls. According to the data of immunoblotting, levels of GFP-AaAtg8-I and GFP-AaAtg8-II showed a pattern similar to that of endogenous AaAtg8-I and AaAtg8-II under each treatment (Fig. 6A). This result suggested that, similar to endogenous AaAtg8, GFP-AaAtg8 was also a useful marker of autophagy in C6/36 cells. When analyzed by fluorescence microscopy, a few fluorescent puncta were observed in mock treated C6/36 cells, indicating a basal level of autophagy in C6/36 cells. DMSO treatment did not change the number of fluorescent puncta. Compared to that in DMSO treated C6/36 cells, the number of fluorescent puncta was decreased in 3-MA treated C6/36 cells whereas increased in Rapa treated C6/36 cells. In CQ treated C6/36 cells, the number of fluorescent puncta was higher than that in DMSO or Rapa treated C6/36 cells. Moreover, the number of fluorescent puncta in C6/36 cells increased when cells were treated with Rapa plus CQ compared to the treatments with Rapa or CQ alone (Figs. 6B and 6C). The above data showed that the number of GFP-AaAtg8 puncta was positively correlated with the abundance of intrinsic AaAtg8-II and GFP-AaAtg8-II detected by immunoblotting analysis. Thus, similar to endogenous AaAtg8, GFP-AaAtg8 could be used to detect autophagy in C6/36 and monitoring GFP-AaAtg8 via immunoblotting and fluorescence microscopy were applicable methods to evaluate the levels of autophagy in C6/36 cells.

**Figure 7:**
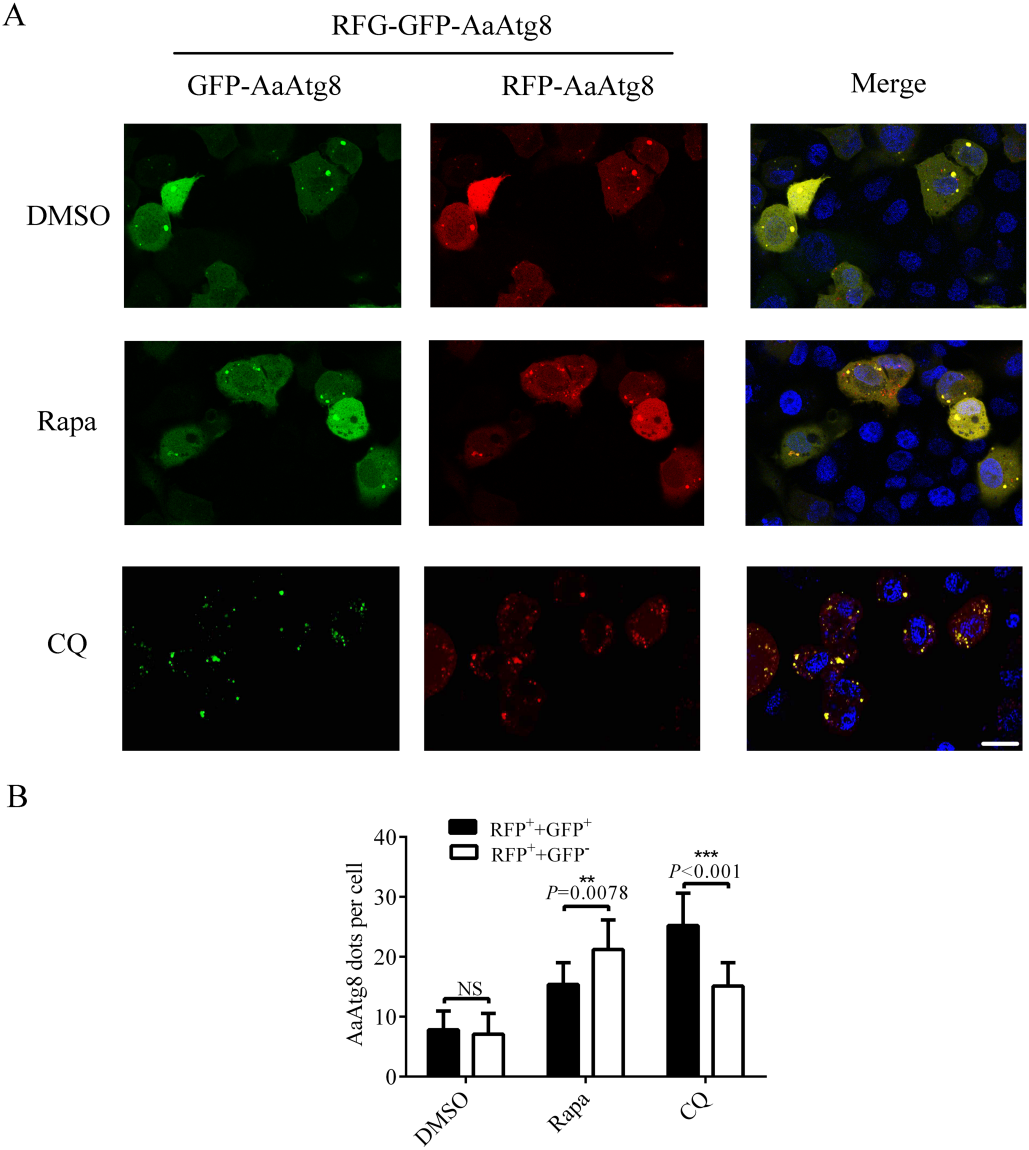
RFP-GFP-AaAtg8 could be used to measure complete and incomplete autophagy in C6/36 cells. C6/36 cells were transfected with RFP-GFP-AaAtg8 plasmid for 12 h and then treated with Rapa or CQ for 6 h and 36 h, respectively. DMSO treated C6/36 cells were used as controls. Cells were observed and cell images were taken under fluorescence microscope (A). The average number of AaAtg8-labeled vacuoles counted by ImageJ in 10 cells was graphed (B). Hoechst 33342 was used to stain nuclear DNA. The statistical significance of data was calculated by *t* test, *P < 0.05; **P < 0.01, NS, not significant. Scale bar: 10 µm.

Since GFP protein is not stable in low pH condition, GFP fluorescence will be quenched after autophagosomes fuse with lysosomes. So, Atg8 is usually fused with GFP plus RFP (a fluorescent protein which is stable in autolysosomes) to monitor autophagy by fluorescence of Atg8. RFP-GFP-Atg8 is observed as yellow signals generated by merged RFP and GFP when associated with autophagosomes. However, the fluorescence of RFP-GFP-Atg8 will be observed as red signals generated by RFP when associated with acidic autolysosomes (Kimura, Noda & Yoshimori, 2007). Thus, if the maturation of autophagosomes into autolysosomes is blocked, yellow puncta will be accumulated without a concomitant increase in red puncta. If autophagosomes can fuse with lysosomes to form autolysosomes, the signal of yellow puncta will decrease, while the signal of red puncta will increase. Thus, monitoring the number of yellow and red puncta provides a convenient and widely used approach to distinguish autophagosomes and autolysosomes in autophagic flux. In order to determine whether this method could be used in C6/36 cells, RFP-GFP-AaAtg8 overexpressing C6/36 cells were treated with Rapa or CQ and RFP-GFP-AaAtg8 was detected by a confocal fluorescence microscopy. DMSO treated C6/36 cells were used as a control. The data indicate that the red and yellow puncta were increased in the Rapa or CQ treated C6/36 cells compared to that in DMSO treated C6/36 cells. However, the number of yellow puncta was lower than that of red puncta in Rapa treated C6/36 cells but higher in CQ treated C6/36 cells. These results suggested that RFP-GFP-AaAtg8 could be used to measure complete autophagy induced by Rapa treatment and incomplete autophagy induced by CQ treatment (Figs. 7A and 7B). All the above data indicated that RFP-GFP-AaAtg8 could be used to monitor flux of autophagy in C6/36 cells.

### Starvation could induce autophagy in C6/36 cells

**Figure 8:**
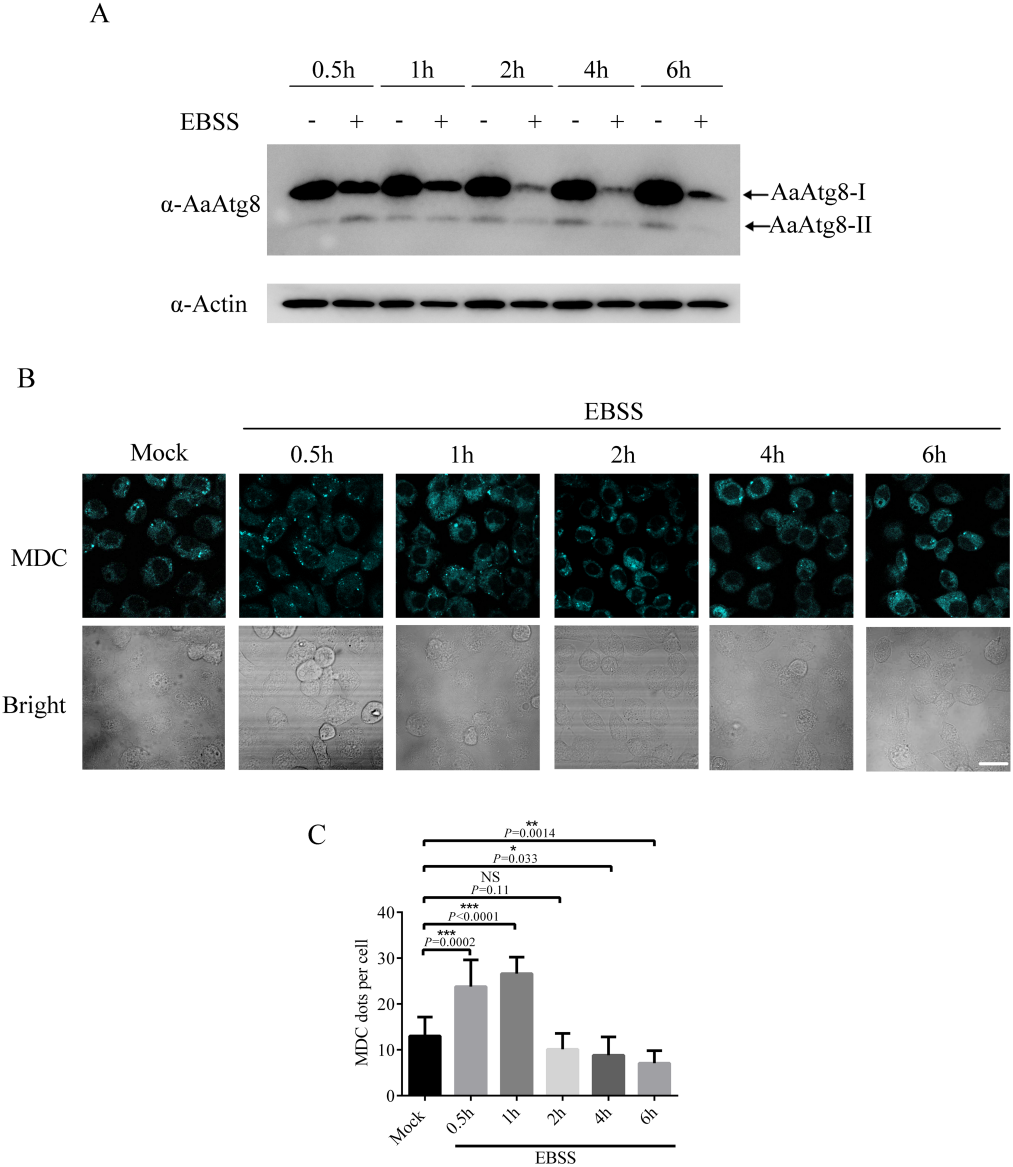
Starvation treatment induced autophagy in C6/36 cells. (A) C6/36 cells were harvested and subjected to immunoblotting using antibody against AaAtg8 after treated with or without EBSS for different time period as indicated. (B) C6/36 cells were subjected to MDC staining after treated with EBSS for different time period and the average number of MDC-labeled vacuoles counted by ImageJ in 10 cells was graphed (C). The statistical significances were calculated by *t* test, *P < 0.05, **P < 0.01, ***P < 0.001, NS, not significant. Scale bar: 10 µm.

In addition to the Rapa treatment, starvation is also frequently used to induce autophagy (Mizushima, Yoshimori & Levine, 2010). Thus, it would be of interest to know whether starvation could induce autophagy in C6/36 cells. Our result revealed that starvation could induce autophagy in C6/36 cells because starvation increased the levels of AaAtg8-II at 0.5 h after EBSS treatment (Fig. 8A). Interestingly, the levels of AaAtg8-II decreased from 1 h to 6 h post starvation treatment. Moreover, C6/36 cells were stained by MDC after EBSS treatment. It was obviously observed that the number of fluorescent puncta was increased at 0.5 h and 1 h after EBSS treatment but decreased from 2 h to 6 h post EBSS treatment (Fig. 8B). These results proved that starvation could induce autophagy in C6/36 cells, but C6/36 cells responded to starvation very rapidly.

## Discussion

Autophagy is an evolutionarily conserved pathway in which intracellular substances can be sequestered within double membrane-bound autophagosomes and targeted to autolysosomes for degradation. Atg proteins play the key roles in autophagy and identification of conserved autophagy genes has permitted genetic and functional dissection of this pathway. In this study, we identified AaAtg8 from *Aedes albopictus* C6/36 cells. It is known that there is only one kind of Atg8 in yeast while there are two subfamilies of Atg8 in mammal, namely, LC3 and GABARAP. It has been reported that the sequence and structure of Atg8 from both *D. melanogaster* and *Bombyx mori* show higher similarity to GABARAP (Hu et al., 2010; Mulakkal et al., 2014). Here, sequence analysis showed that AaAtg8 also had the higher sequence similarity to GABARAP subfamily (Figs. 1 and 2).

There are reports showed that autophagy is an integral part of developmental processes, such as dauer formation in nematodes and metamorphosis in fruit flies (Melendez et al., 2003; McPhee & Baehrecke, 2009). During *Drosophila* metamorphosis, larval tissues (midgut, salivary gland, and fat body) undergo autophagic degradation (Rusten et al., 2004; Berry & Baehrecke, 2007; Denton et al., 2009). In female *Aedes aegypti* mosquitoes, autophagy functions in maintaining egg maturation cycles (Bryant & Raikhel, 2011). These researches illustrate that autophagy plays a role in the life of insect from embryonic development to maturation. This study found that in *Aedes albopictus*, there was a ubiquitous expression of AaAtg8 in all developmental stages (Fig. 3). Moreover, transcription levels of AaAtg8 in female adults were significantly higher than that in male adults. These results implied that AaAtg8 played a role in the developmental and adult stages of *Aedes albopictus*. Further investigations are needed to elucidate this subject.

This is the first work to suggest that Rapa and starvation treatments could induce autophagy successfully in C6/36 cells. It is noteworthy that C6/36 cells responded to starvation very rapidly (Fig. 8). This study also illustrated that 3-MA and CQ provided useful ways to inhibit early and late stages of autophagy, respectively. Both 3-MA and CQ are commonly used autophagy inhibitors, but they have different impacts on the turnover of AaAtg8. 3-MA inhibits PI3-kinase which functions in early stage of autophagy, while CQ blocks the fusion of autophagosomes and lysosomes (Ha & Kim, 2016). So, 3-MA treatment suppresses the generation of Atg8-II, while CQ treatment results in accumulation of Atg8-II. These effects can explain the data obtained in the case of 3-MA or CQ treatment in this study (Figs. 4–6).

Although various methods for monitoring autophagy had been used in some cell lines and organisms, the methods to detect autophagy in *Aedes albopictus* have not been reported so far. This study tested several methods to monitor autophagy in C6/36 cells for the first time. First, we proved that the MDC staining could reflect autophagy in C6/36 cells (Fig. 4). Second, autophagy of C6/36 cells also could be evaluated by monitoring the conversion of AaAtg8-I to AaAtg8-II (Fig. 5). Finally, turnover of fluorescent AaAtg8 assay (including GFP-AaAtg8 and RFP-GFP-AaAtg8) could trace the autophagic flux in C6/36 cells (Figs. 6 and 7). Even though both GFP-AaAtg8 and RFP-GFP-AaAtg8 could be used to monitor flux of autophagy in C6/36 cells, RFP-GFP-AaAtg8 was capable of analyzing real-time state of autophagosomes and lysosomes fusion, thus allowing more precise analysis of autophagy flux.

## Conclusions

We identified an Atg8 homolog from *A. albopictus* and named it AaAtg8. Sequence analysis revealed that AaAtg8 protein was highly conserved in Atg8 family. There was a ubiquitous expression of AaAtg8 during developmental and adult stages of *Aedes albopictus*. Additionally, our data proved that AaAtg8 was a functional Atg protein of *Aedes albopictus*. Moreover, the study established the basic methods for studying autophagy in *Aedes albopictus* C6/36 cells. First, this work proved that both rapamycin and starvation can induce autophagy in C6/36 cells, and that 3-methyladenine and chloroquine could be used to inhibit early and late stages of autophagy in C6/36 cells, respectively. Second, the work demonstrated that the level of autophagy in C6/36 cells could be monitored by the turnover assay of AaAtg8 or fluorescent AaAtg8. Also, the results in this study showed that MDC staining could be used to detect autophagy in C6/36 cells. We believe that our work would facilitate an understanding of the autophagic mechanisms in the *A. albopictus* mosquito.

##  Supplemental Information

10.7717/peerj.5988/supp-1 Data S1 Supplemental raw data

10.7717/peerj.5988/supp-2 Figure S1 Supplemental WB figures

10.7717/peerj.5988/supp-3 Supplemental Information 1 The sequence information of AaAtg8

## Abbreviations

3-MA: 3-methyladenine
AaAtg8 (or *Aaatg8*): *Aedes albopictus* Atg8 (or *atg8*)
*Aas7*: *Aedes albopictus* ribosomal protein *s7*
Atg: autophagy-relate gene
bp: base pair(s)
cDNA: DNA complementary to RNA
CHIKV: chikungunya virus
CMA: chaperone mediated autophagy
CQ: chloroquine
DENV: dengue virus
DNase: deoxyribonuclease
EBSS: Earle’s Balanced Salt Solution
FBS: Fetal Bovine Serum
GABARAP: γ-aminobutyric acid (GABA)-receptor-associated protein
GABARAPL: γ-aminobutyric acid (GABA)-receptor-associated protein like
GATE-16: Golgi-associated ATPase enhancer of 16 kDa
HRP: horseradish peroxidase
LC3: light chain 3
MDC: monodansylcadaverine
MEM: Minimum Essential Media
Min: minute(s)
mRNA: messenger RNA
ORF: open reading frame
PBS: phosphate buffered saline
PCR: polymerase chain reaction
PE: phosphatidylethanolamine
PI3K: phosphatidylinositol 3-kinases
PI3P: phosphatidylinositol 3-phosphate
qPCR: quantitative real-time PCR
RACE: rapid amplification of cDNA ends
Rapa: rapamycin
SDS-PAGE: sodium dodecyl sulfate polyacrylamide gel electrophoresis
TBST: Tris Buffered Saline with Tween-20
ULK: unc-51 like autophagy activating kinase
*UTR*: untranslated region

## Additional Information and Declarations

### DNA Deposition

The following information was supplied regarding the deposition of DNA sequences: The AaAtg8 sequence described is available in GenBank, accession number MH243747.

### Data Availability

The following information was supplied regarding data availability: The raw data are provided in the Supplemental Files.
