# Supplementary figures and images for: Identification of AaAtg8 as a marker of autophagy and a functional autophagy-related protein in Aedes albopictus
Source: PeerJ. 2018 Nov 21;6:e5988. doi: 10.7717/peerj.5988 (PMC6252070; doi:10.7717/peerj.5988)

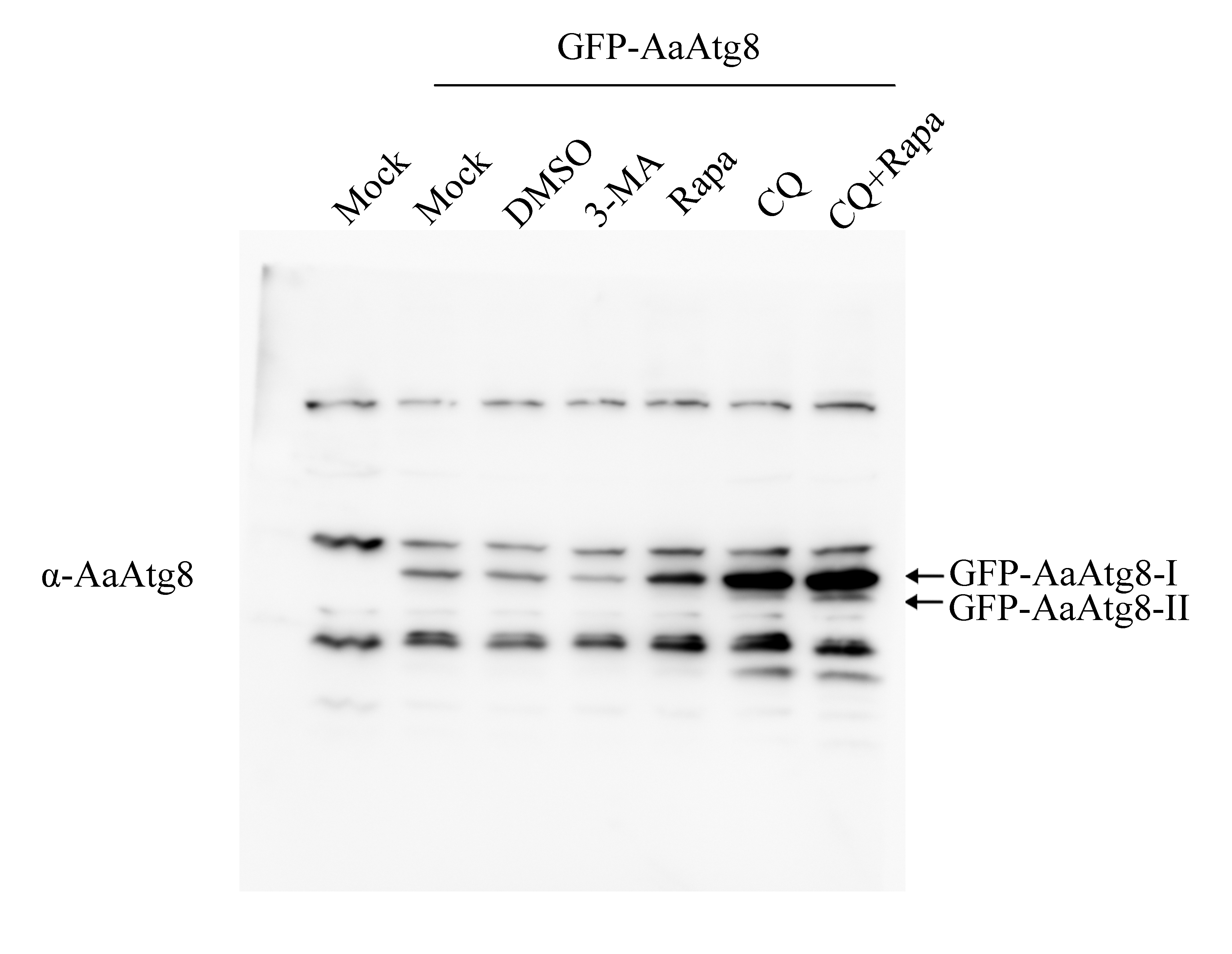

Supplement: Figure S1 [file peerj-06-5988-s002.zip › Supplemental AaAtg8 S3.png]

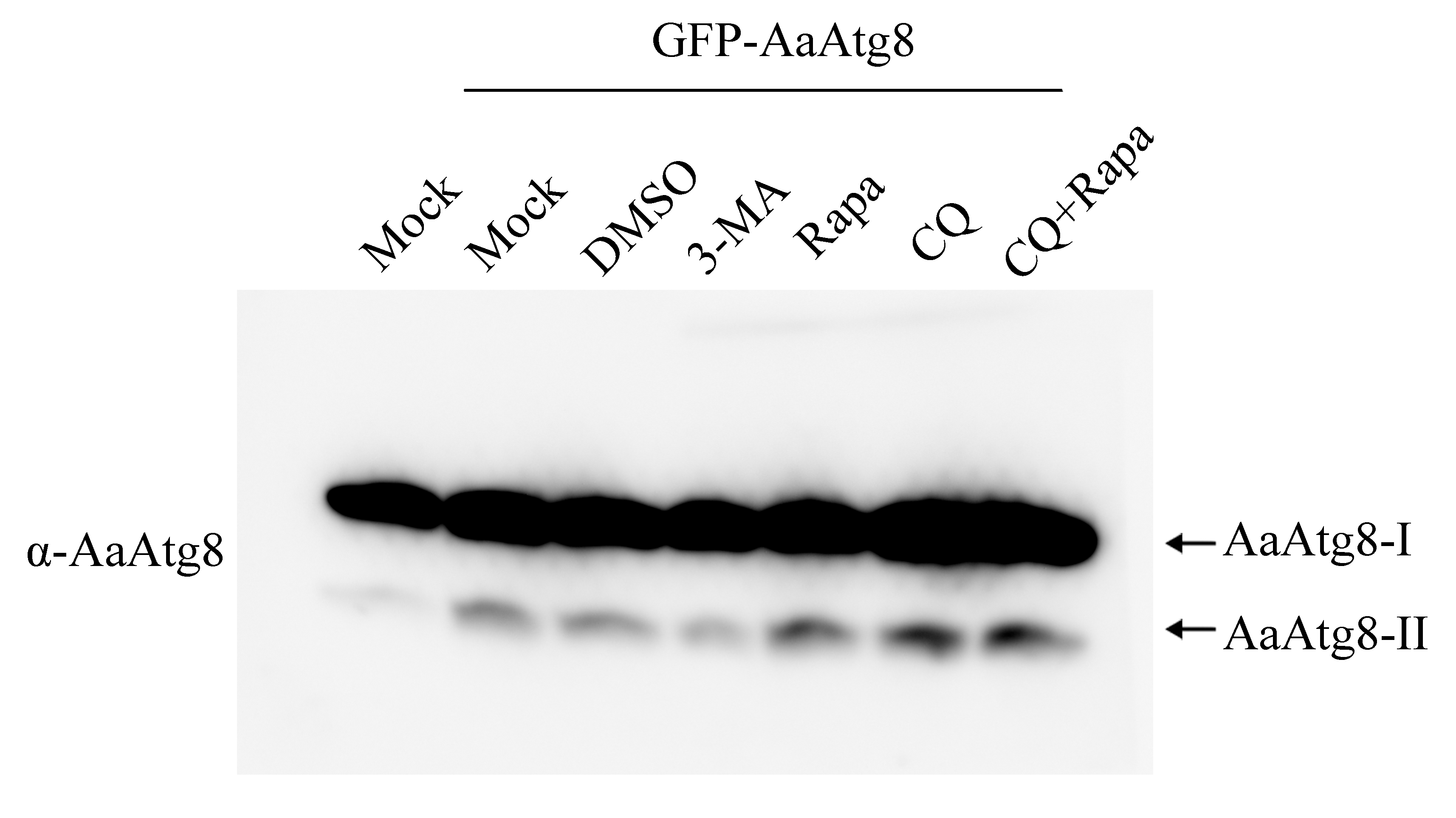

Supplement: Figure S1 [file peerj-06-5988-s002.zip › Supplemental AaAtg8 S4.png]

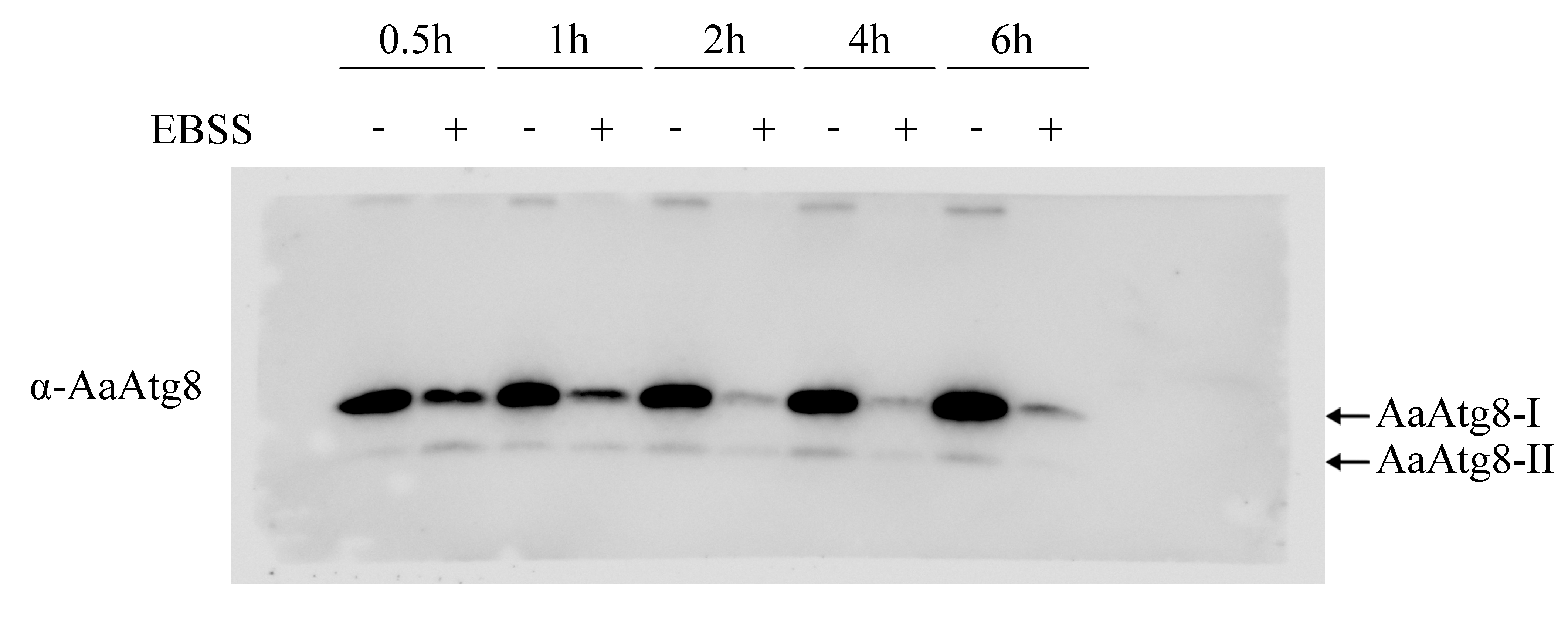

Supplement: Figure S1 [file peerj-06-5988-s002.zip › Supplemental AaAtg8 S7.png]

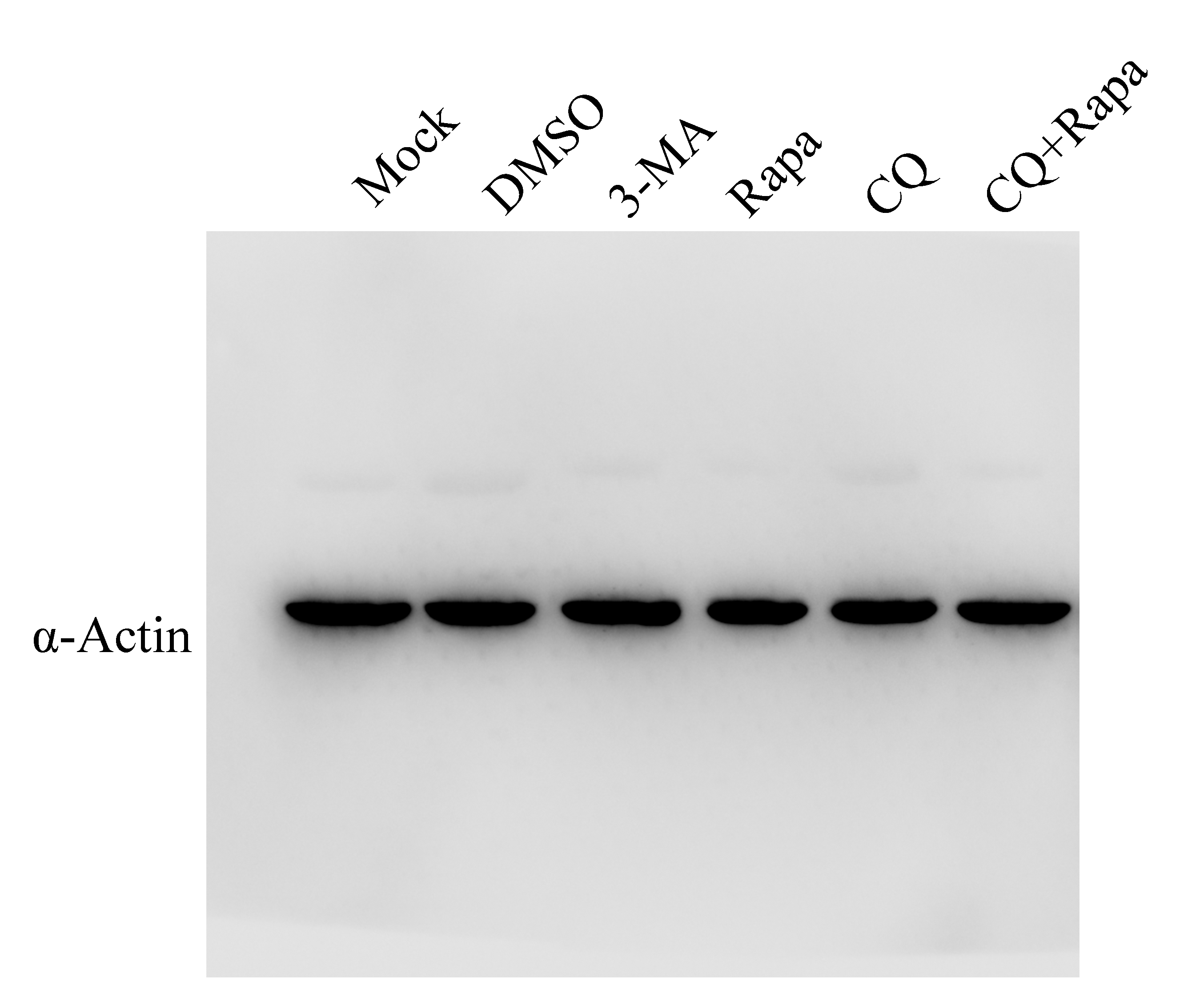

Supplement: Figure S1 [file peerj-06-5988-s002.zip › Supplemental Actin S2.png]

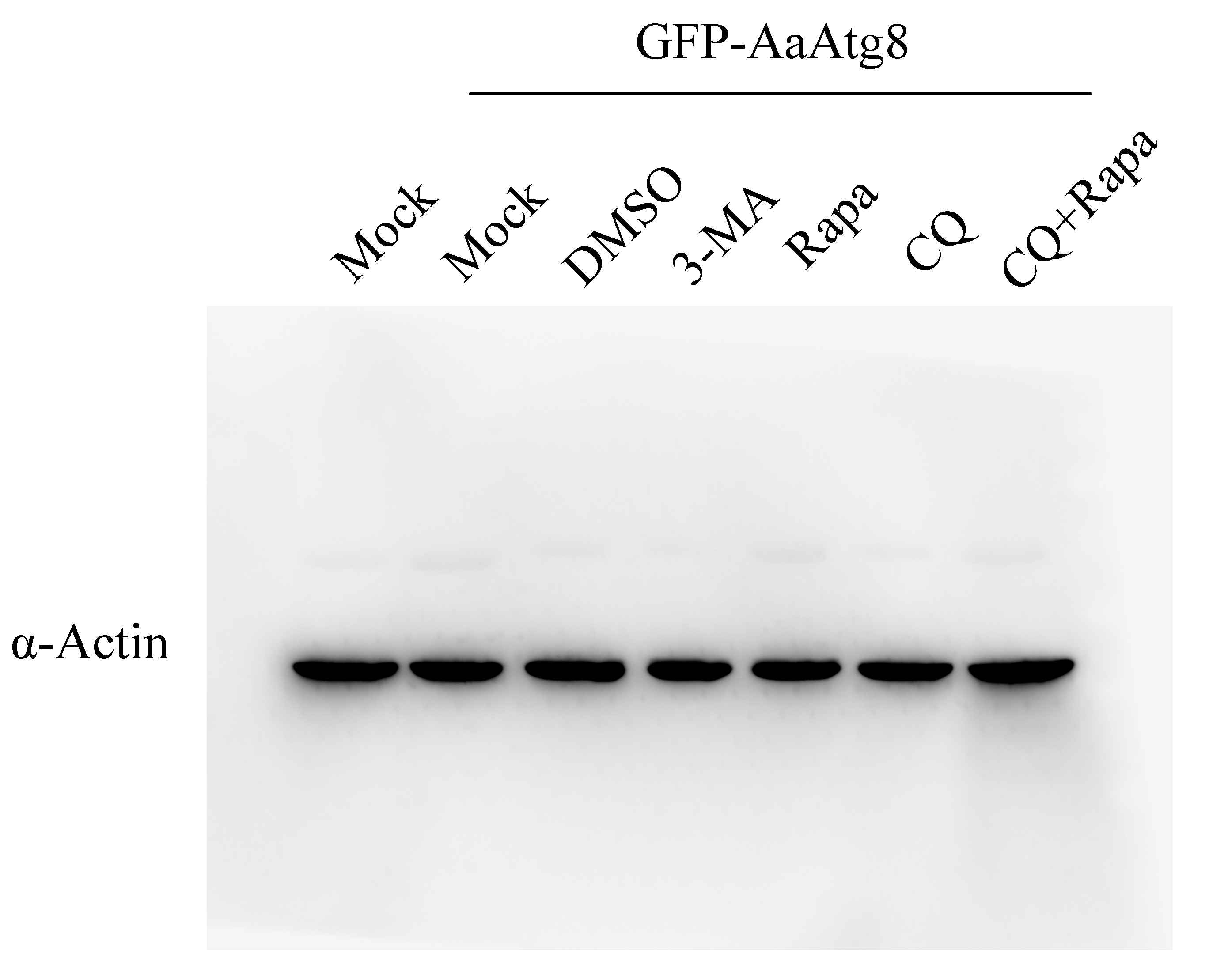

Supplement: Figure S1 [file peerj-06-5988-s002.zip › Supplemental Actin S6.png]

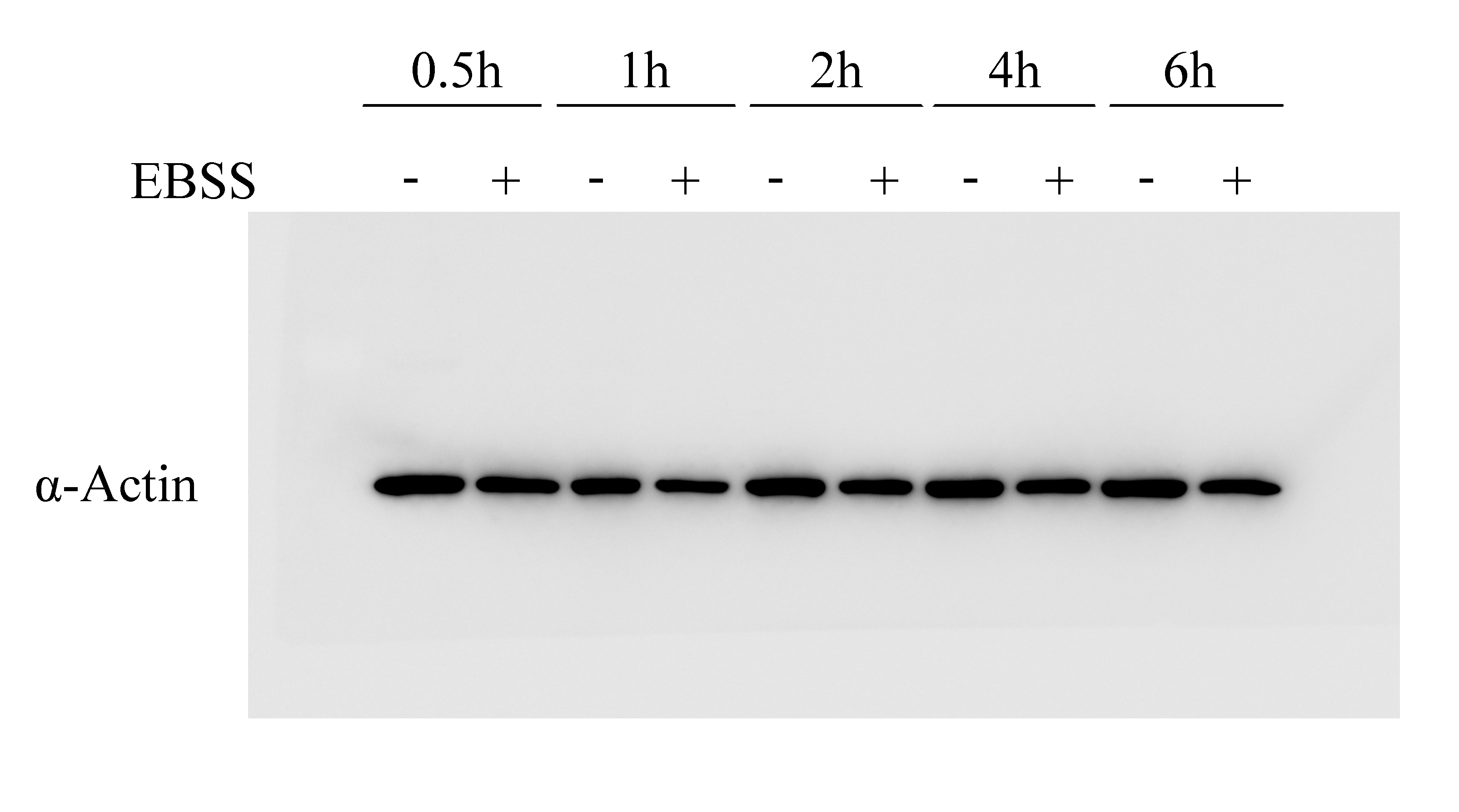

Supplement: Figure S1 [file peerj-06-5988-s002.zip › Supplemental Actin S8.png]

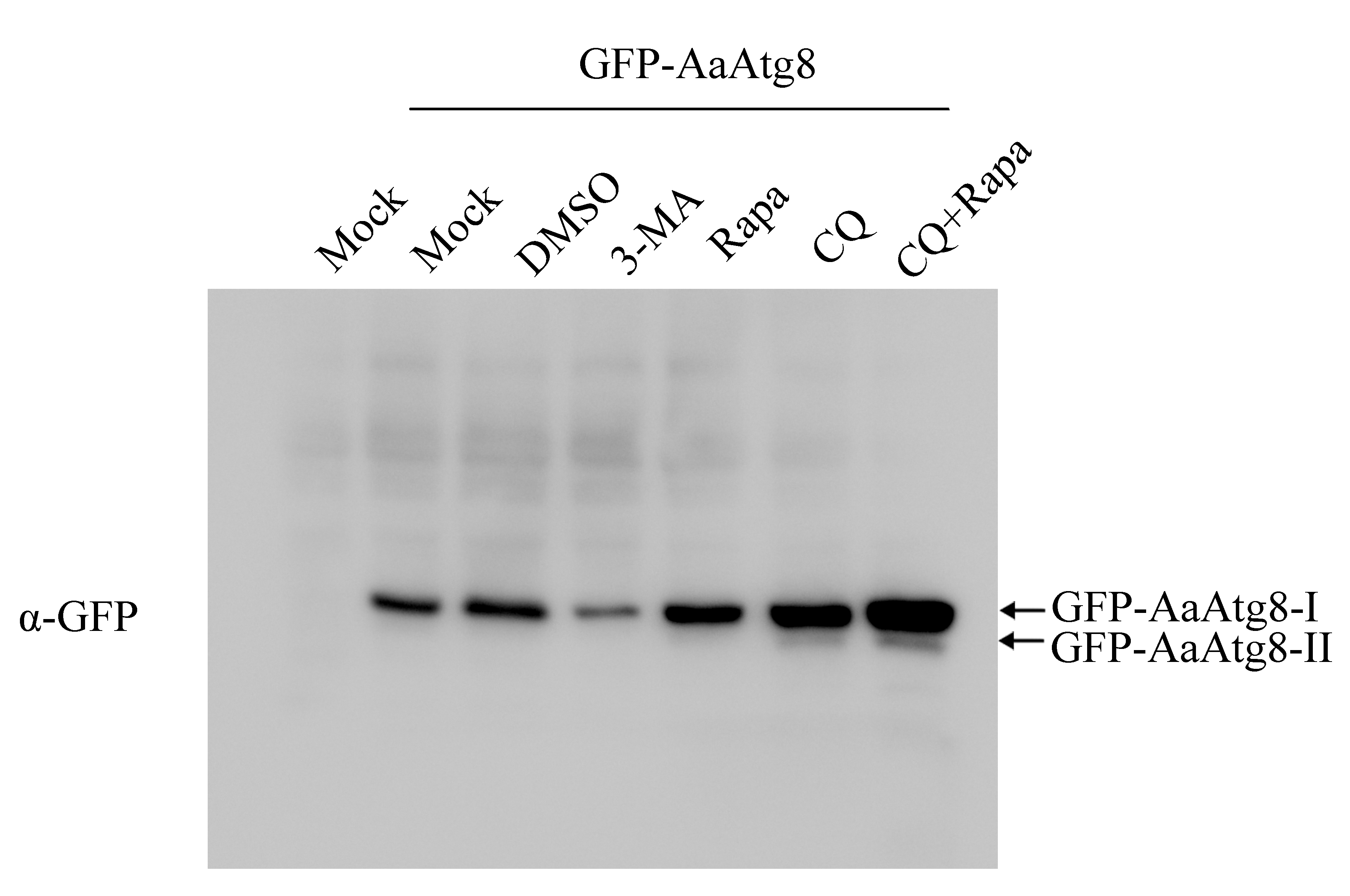

Supplement: Figure S1 [file peerj-06-5988-s002.zip › Supplemental GFP S5.png]

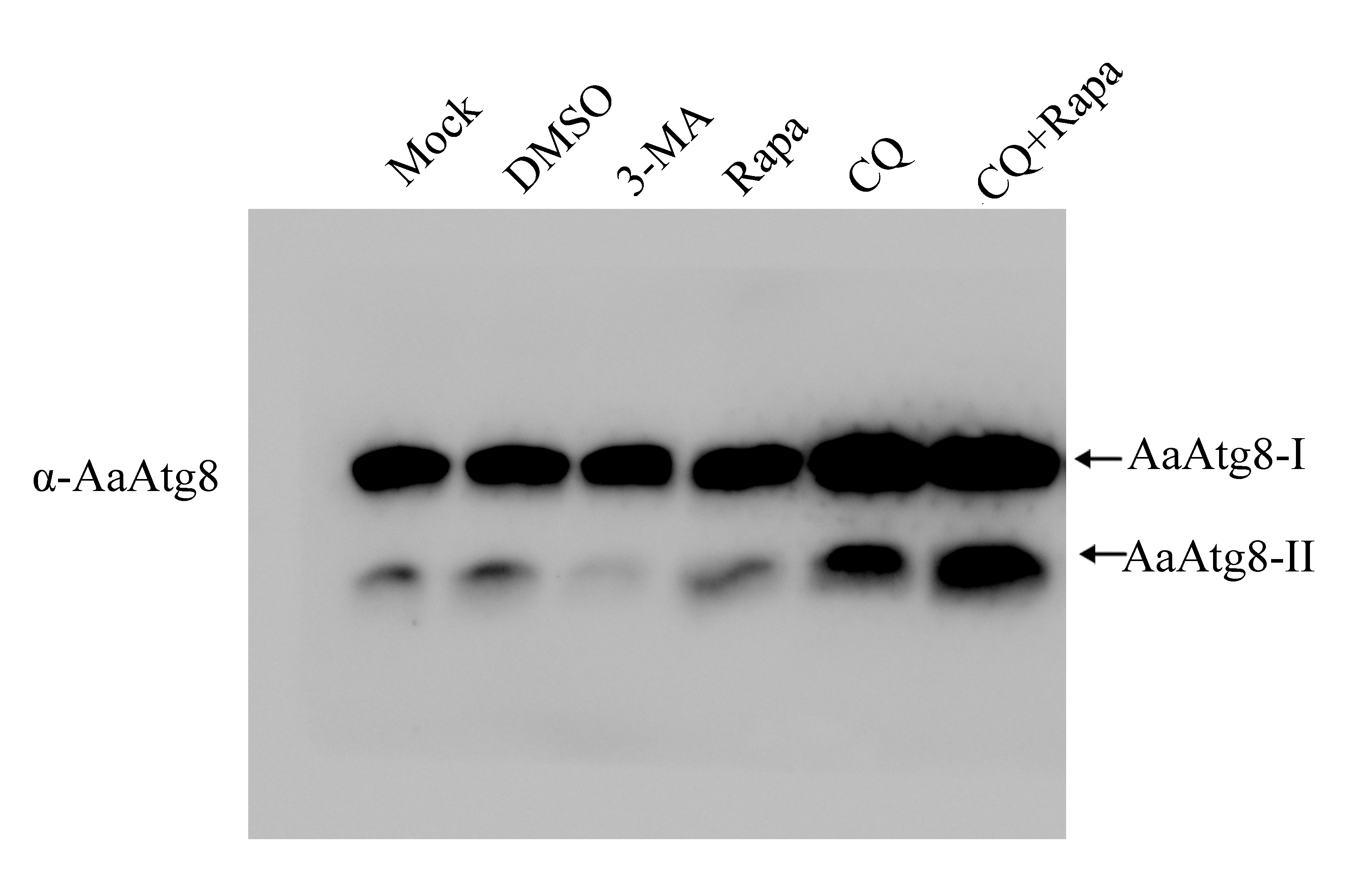

Supplement: Figure S1 [file peerj-06-5988-s002.zip › Supplemental AaAtg8 S1.png]
