# Supplementary material for: Identification of AaAtg8 as a marker of autophagy and a functional autophagy-related protein in Aedes albopictus
Source: PeerJ. 2018 Nov 21;6:e5988. doi: 10.7717/peerj.5988 (PMC6252070; doi:10.7717/peerj.5988)
Supplement: Supplemental Information 1 [file peerj-06-5988-s003.pdf]

GenBank accession number: MH243747

Aaatg8(ORF=357bp)

1 CTAATACGACTCACTATAGGGCAAGCAGTGGTATCAACGCAGAGTACATGGGGAGTTTCA
61 GTGAAAACATCGTCGAGAACACATCTGTTTAGTGGATAACGCTCCTGATAATTTTGATAA
121 GAGTCAACACAAGAAAAAAGCTAGTGCAAAA**ATG**AAATTTCAATACAAGGAAGAACACCC
181 CTTCGAGAAGCGAAAGGCCGAGGGAGATAAAATCCGACGCAAATACCCGGAACGAGTACC
241 CGTGATTGTTGAGAAAGCTCCCAAGGCTCGCATTGGAGATTTGGATAAGAAGAAGTATCT
301 AGTCCCGTCCGACTTGACCGTCGGCCAGTTCTATTTCCTAATCCGCAAGAGGATTCACCT
361 AAGGCCTGAGGATGCCCTGTTCTTCTTCGTTAACAATGTAATTCCACCAACATCGGCAAC
421 AATGGGCTCGCTGTACCAGGAACACCACGAGGAGGACTACTTCCTCTATATTGCTTATTC
481 TGATGAGAACGTGTATGGAAACAAG**TAA**ATAACGAGAGTGATATGTATAGAAAAGGACTG
541 CTATATATGTATTTGCTATATGATATTTCCTGTTCGTTTTGACAATATTATTTTGCTGCA
601 CGCTTTCGGGTAGATATGTTCCAGCGATAAAGTTTGTTAGGTTACAGCGACACCGACATC
661 CGTTAACGTTTCACCGCGTTACGATGCATTTGAAACATGGTTCCAGCATCTATTCCGGTA
721 TATGAATGTCGAATGAAAGGCAGAACAACATTTCAACAAGTATTGTAACCGAATTGAAAT
781 ACAAACAGCTAACATAAGTCAAATTAGTTTAATTTGTTAGTGCTGGCTTGTCATTTTAAG
841 TGCTTGTTGAAAAGGATCTGTGCAGTCTACAAATTGGTATTTGAAGAACTTGTATAACAA
901 GATTCTCGTGTTTATTTTGTGTATTTGTATAATTTGTCTGCGCCATATACCGGAATAGAC
961 ACATCTAGAAATATTTCTCAACACAGTCACCATGTGTATGTTTCAAAACTAACAATTGTA
1021 GTAAACGCAAATGCATATTGAATGAAGAGCATACATAAACCTATATAAAAAAGTGAATAT
1081 TATTCAAAACGTAATAAAAAAACACATAAATACTGCAAAATGTGTGAAAGCACATACCAA
1141 AAACACATTTTTAAACATAGTTTCTCATTTTTAAATATACTAGTAACTCCAATCTGTATT
1201 TTCTTCGTTTAATTTACACAGATATATGGAATAAATTATAAGTCGTAAGTAGGTCAAAAA
1261 AAAAAAAAAAAAAAAAAA
